# An investigation into structural behaviors of skulls chewing food in different occlusal relationships using FEM

**DOI:** 10.1002/cre2.273

**Published:** 2019-12-20

**Authors:** Yeo‐Kyeong Lee, Youn‐Sic Chun

**Affiliations:** ^1^ Architectural and Urban System Engineering, Division of Sustainable Systems Engineering, ELTEC College of Engineering Ewha Womans University Seoul South Korea; ^2^ Orthodontics & dentofacial orthopedics, School of Medicine Ewha Womans University Seoul South Korea

**Keywords:** chewing simulation, FEA (finite element analysis), occlusal relationship, skull, structural behavior

## Abstract

**Objectives:**

This study aims to investigate the effect of different occlusal relationships on skull structural and mechanical behaviors through simulation of chewing food.

**Methods:**

Finite element (FE) skull models of occlusion for Class I, end‐on Class II, and full‐cusp Class II were generated. End‐on Class II and full‐cusp Class II were chosen as mild and severe Class II occlusions, respectively. A simplified food bolus was introduced between the upper and lower dentition of the right molars. Chewing food was simulated in the skulls by moving the mandible. An experiment was conducted to measure strains at selective locations and compared them to the analytical results for validation.

**Results:**

In the early stages of mandibular movement, masticatory forces predicted from the skull models without food were lower than the skull models with food but increased drastically after occluding teeth full enough. As a result, the relationship between masticatory force and mandible movement shows that there is no significant difference between the skull models with food and without food in the range of human masticatory force, approximately 250 N. In all the cases of skulls including a food bolus, stress was similarly propagated from the mandible to the maxilla and concentrated in the same regions, including the mandibular notch and alveolar bone around the lower molars.

**Conclusion:**

It is predicted that there is no significant difference of bite force–mandible movement relationships and stress distributions of skull and teeth, between end‐on Class II and full‐cusp Class II models. When simulating chewing activities on candy and carrot, it is also found that there is no difference of masticatory performance between Class II occlusions, from structural as well as mechanical perspectives.

## 1 INTRODUCTION

According to the Korean Health Insurance Review & Assessment Service, orthodontic patients have been steadily increasing owing to malocclusion treatment and aesthetic functions. During orthodontic treatment, extraction is generally done to retain teeth spacing and straighten teeth arrangement, which may cause changes of occluding and masticatory activities. Therefore, it is crucial to predict the differences in skull structural and mechanical behaviors according to different occlusal relationships.

Finite element (FE) analysis has been widely used to simulate different behaviors in dentofacial orthopedics and dental biomechanics (Ammar, Ngan, Crout, Mucino, & Mukdadi, 2011; Borcic et al., 2005; Cattaneo, Dalstra, & Melsen, 2005; Chang, Shin, & Baek, 2004; Field et al., 2009; Geramy & Morgano, 2004; Hsu, Chen, Chen, Huang, & Chang, 2009; Kibi et al., 2009; Lee, Choi, Lee, Ahn, & Noh, 2018; Liang, Rong, Lin, & Xud, 2009; Liu, Chang, Wong, & Liu, 2012; Rudolph, Willes, & Sameshima, 2001; Singh, Mogra, Shetty, Shetty, & Philip, 2012; Soares et al., 2014; Toms & Eberhardt, 2003; Vukicevic, Zelic, Jovicic, Djuric, & Filipovic, 2015; Yu, Baik, Sung, Kim, & Cho, 2007; Zhang, Cui, Lu, & Wang, 2017), because of many problematic issues in clinical testing, such as subject discomfort and limit of accessible areas. In the study conducted by Lee et al. (2018), FE analysis was performed to investigate the mechanical effect of different protrusion positions of a mandibular advancement device (MAD) on teeth and facial bones, since the MAD‐induced stress and strain have not been measured directly from living structures. Ammar et al. (2011) used the FE jawbone model to simulate canine retraction with mini‐screw anchorage and compared stresses on the mini‐screw and fracture failure of tangential orthodontic forces. Zhang et al. (2017) constructed a mandibular first molar FE model and simulated various occlusal load conditions through area size, location, and direction of loading. Rudolph et al. (2001) and Cattaneo et al. (2005) also performed FE analyses of a 1 or 2 teeth model applying various orthodontic tooth movements and evaluating stress at the tooth, periodontal ligament (PDL), and alveolar bone. Liang et al. (2009) generated FE models of the maxilla and maxillary incisors and reported that teeth stress and strain distributions change depending on the orthodontic method; horizontal retraction force, intrusive vertical force, and lingual root torque. In the study conducted by Soares et al. (2014), the biomechanical behaviors of maxillary premolar teeth were estimated using FE analysis and strain gauge testing. They compared the predicted stress distributions regarding tooth root morphology and abfraction depth.

Relatively little research has been conducted to investigate occlusal behaviors from the full skull FE model. In the previous studies (Lee, Kim, & Park, 2017; Lee, Park, & Kim, 2016), FE modeling methods for simulation of occluding teeth in full‐skull were proposed and parametric studies of boundary and loading conditions were performed through FE analysis to find out how to prescribe proper support and loading in the full skull model. Simulation is possibly much closer to actual activity using the full skull model, and the stress and strain distributions from the mandible to the maxilla can be predicted. Moreover, it can minimize the effect of modeling error, such as occlusal surface modeling, as one can investigate the structural behaviors of the skull from a more comprehensive overall viewpoint.

However, from a clinical viewpoint, it is imperative to know what happens mechanically in the skull and teeth when chewing food rather than just occlusion. Therefore, this paper aims to investigate the effect of different occlusal relationships on the structural and mechanical behaviors of skulls, such as forces and stress distributions through simulation of chewing food.

## 2 MATERIALS AND METHODS

### 2.1 Generation of FE model

**Figure 1:**
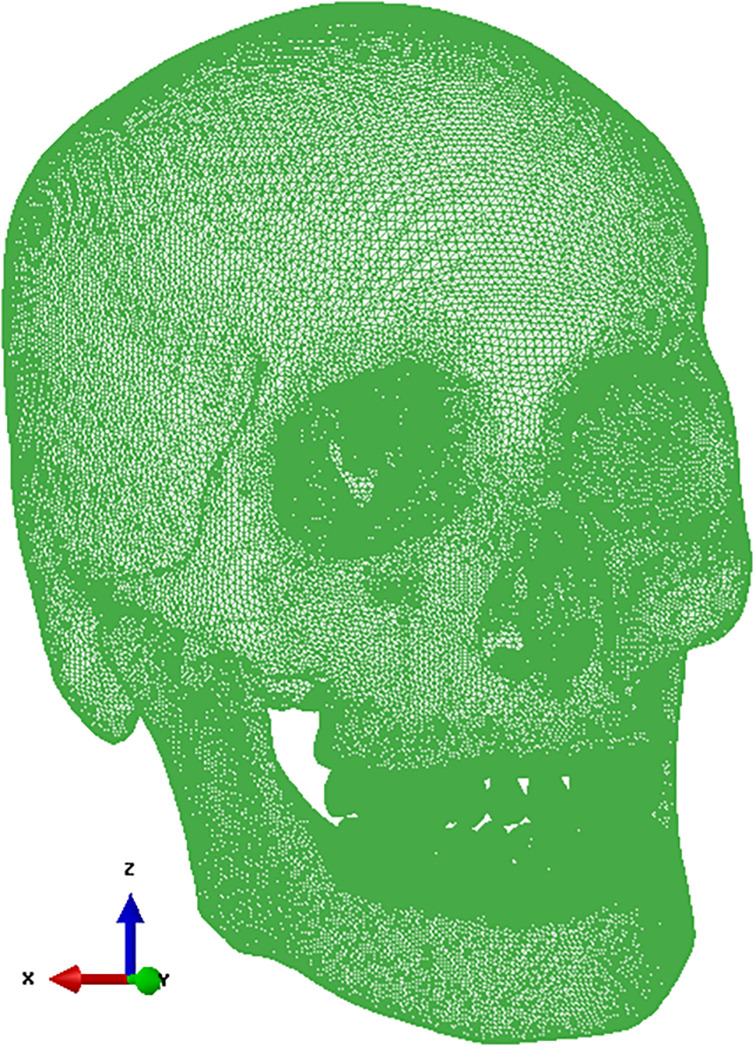
Finite element (FE) skull model with occlusion for Class I

CT images (SOMATOMTM SENSATION, Siemens AG, Germany, 120 kVp, 200 ms, 0.75 mm‐thickness) were captured from a 38‐year‐old male skull with normal occlusion status and constructed as the FE skull model with a Class I occlusion (Figure 1); previously published papers detail the modeling process (Lee et al., 2016, 2017; Seol, 2014). Taking into consideration post‐orthodontic general treatment cases (Janson, Sathler, Fernandes, Zanda, & Pinzan, 2010; Liu & Melsen, 2001), end‐on Class II and full‐cusp Class II were chosen as mild‐Class II and severe‐Class II, respectively. Skull models with Class II occlusions were created by modifying the skull with occlusion Class I. The right upper first premolar (#14) was extracted and the neighboring teeth, second premolar (#15), first molar (#16), and second molar (#17) were moved along a mesial direction to adjust molar relationships. Half of the space created by the extraction of tooth #14 remained in the case of the skull with end‐on Class II occlusion, whereas in the case of the skull with full‐cusp Class II occlusion, that space was filled by the neighboring teeth.

**Figure 2:**
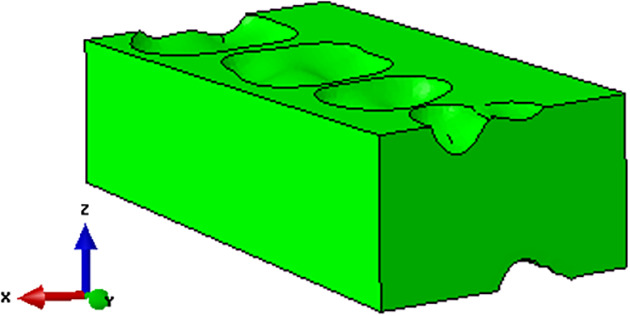
Food bolus cut in the form of the occlusal surface

The food bolus was simplified as a rectangular parallelepiped configuration, and the size determined by considering the range of the molars to be 18 mm × 32 mm × 10 mm (width × length × height) as illustrated in Figure 2. To place the food bolus between the upper and lower dentition, the mandible was moved downward by 5 mm from its initial position. Furthermore, the food bolus was cut in the form of the occlusal surface to have perfect matches between antagonistic teeth and food surfaces. Therefore, the actual distance of the food bolus between antagonistic teeth was approximately 5~6 mm. A total of six FE model cases were generated according to occlusal relationships and simulation types, as seen in Table 1.

**Table 1:** Details of skull models

Model name	Occlusal relationship	Simulation	Teeth arrangement
N_NF	Class I	Teeth occluding	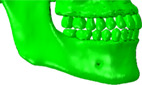
E_NF	End‐on Class II	Teeth occluding	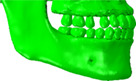
F_NF	Full‐cusp Class II	Teeth occluding	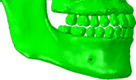
N_IF	Class I	Food chewing	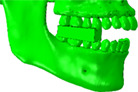
E_IF	End‐on Class II	Food chewing	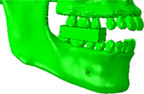
F_IF	Full‐cusp Class II	Food chewing	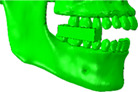

**Figure 3:**
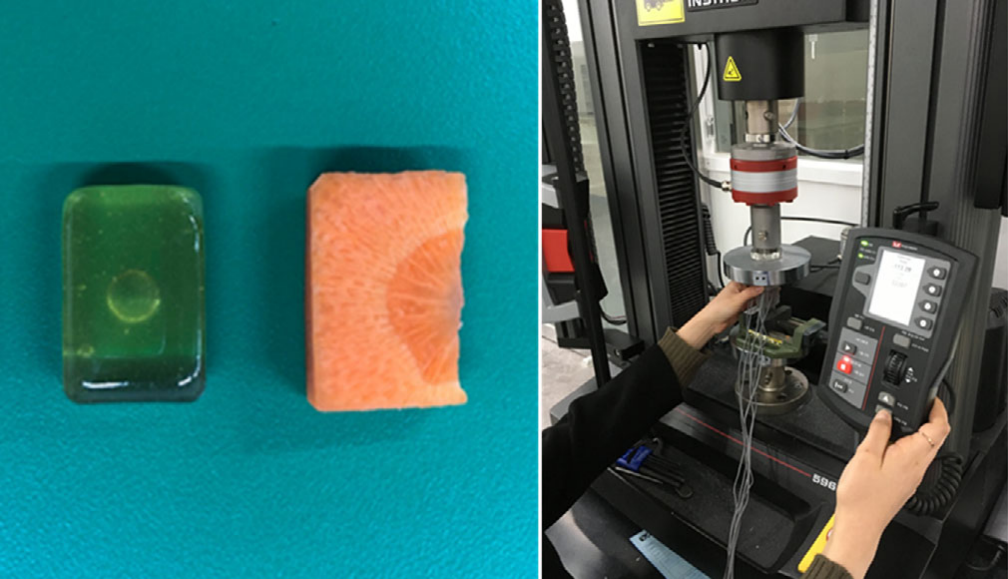
Compressive strength test setup of candy and carrot

**Figure 4:**
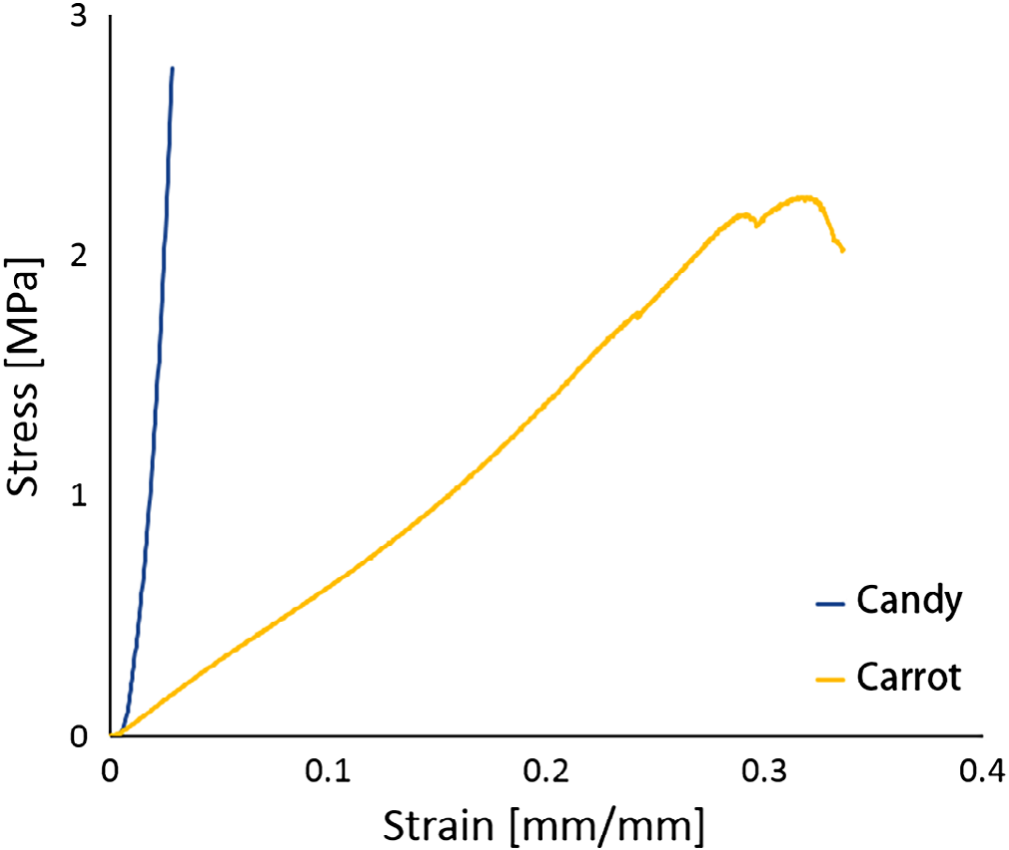
Stress–strain curves obtained from tests of food

The FE models consist of five or six parts; teeth, PDL, cortical bone, cancellous bone, the temporomandibular joint (TMJ) disc, or food bolus; Table 2 outlines element details and the corresponding material properties. Each material property was linear elastic as referred to previous papers (Minch, 2013; Santis, Ambrosio, & Licoais, 2002; Singh & Detamore, 2009). Compressive strength testing was performed with candy and carrot to define the food material model since they are similar in size to the FE food model, 15 mm × 23 mm × 6 mm (width × length × height) as shown in Figure 3. As illustrated in Figure 4, stress–strain curves were obtained from the tests and the candy's linear‐elastic material behavior was applied to the food bolus.

**Table 2:** Element details and material properties of each parts

Part	Number of elements	Size of elements	Elastic modulus	Poisson's ratio
Teeth	569,172~587,766	0.2~0.7 mm	20 GPa	0.3
PDL	64,300~66,844	0.3~1.0 mm	0.75 MPa	0.45
Cortical bone	1,817,511~1,820,741	0.3~3.0 mm	14.5 GPa	0.323
Cancellous bone	879,726~890,403	0.6~2.5 mm	1.37 GPa	0.3
TMJ disc	1,465~1,925	0.8~3.0 mm	21.7 MPa	0.3
Food (candy)	65,161~67,783	0.4~1.2 mm	120.17 MPa	0.2

### 2.2 Simulation of chewing food

The skull models were imported into commercial software ABAQUS ver. 6.10‐3 (Dassault Systemes, Velizy‐Villacoublay, France) to prescribe loading and boundary conditions for chewing simulation. Small sliding contact with a friction coefficient of 0.2 (Wierszycki, Kakol, & Lodygowski, 2006) was established between the upper and lower dentition (two‐body interaction) for the skull models without food; N_NF, E_NF, and F_NF. In cases of the skull including food, N_IF, E_IF, and F_IF, the surfaces between antagonistic teeth and the food bolus (three‐body interaction) were simulated perfect bonding to avoid penetration error in the food model. In both the skull models with food and without food, interfaces between teeth and PDL, between PDL and bone, and between TMJ disc and bone were modeled by sharing nodes to make the connectivity of structural behaviors. In this study, it was assumed that chewing food is primarily driven by mandibular movement in a coronal‐apical direction (Kim, Lee, & Park, 2016; Merdji et al., 2013) and simulated in a single cycle (Martinez Choy, Lenz, Schweizerhof, Schmitter, & Schindler, 2017). Two points were selected at the mandibular surface as loading points taking into consideration the location of the first molar and driving direction of the masseter muscle, which are the keys to masticatory activity. Loading was prescribed in the form of displacement control as the points were moved up 5 mm in the z‐direction and freed in all other directions. The periphery of the foramen magnum was constrained in all translational degrees of freedom to make the skull models statically stable during loading.

## 3 RESULTS

### 3.1 Masticatory force‐mandible movement curves

**Figure 5:**
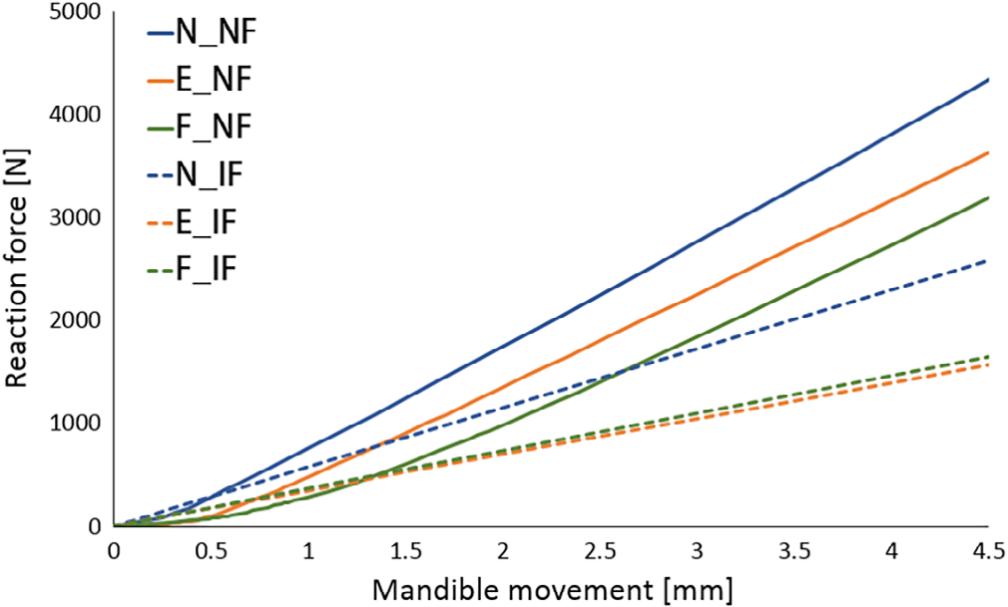
Force–displacement relationships obtained from finite element (FE) analysis

**Figure 6:**
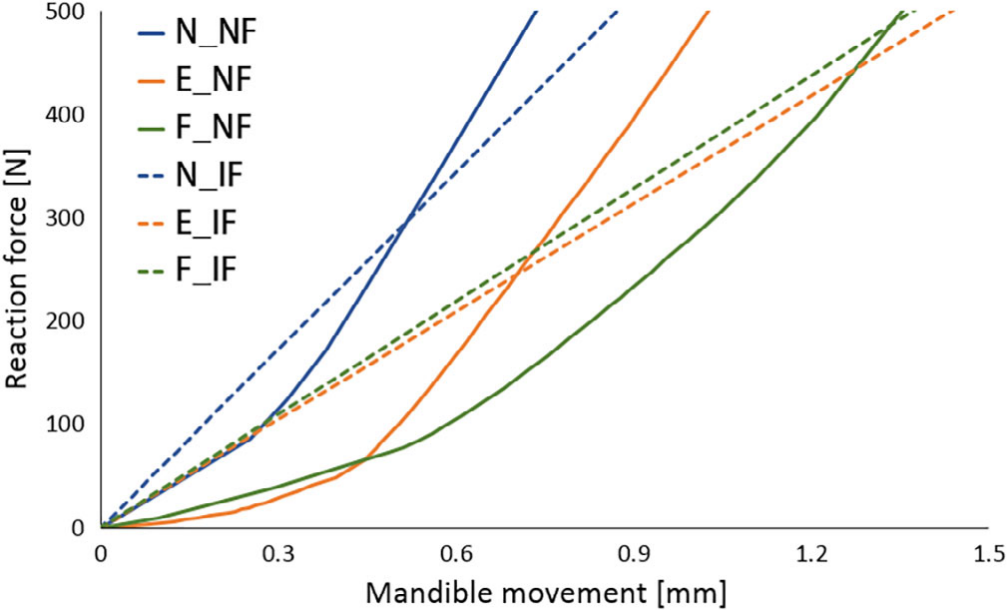
Force–displacement relationships in the range of masticatory force

The first assessment was the relationship between masticatory force and mandible movement in the skull models; masticatory forces were calculated as the sum of reaction forces at the constrained region. Figure 5 shows the force‐displacement curves obtained from the analytical results, and Figure 6 is a part of Figure 5 focusing on the average range of human masticatory force.

The skull models including food exhibit higher masticatory forces than the skull models without food in a very early stage of mandibular movement because the teeth in N_IF, E_IF, and F_IF were in perfect contact with the food bolus from the initial position, while there was a gap between the maxillary and mandibular teeth in N_NF, E_ NF, and F_NF. Skull models without food showed several changes in the slope of force‐displacement with changes in the contact area between the teeth.

After the mandibular movement reached around 1.5 mm, the maxillary and mandibular teeth occluded adequately enough; masticatory force and the slope of the skull models without food drastically increased as shown in Figure 5. An elastic modulus of teeth (20 GPa) was grander than that of food (120.17 MPa); therefore, N_NF, E_NF, and F_NF under occluding teeth simulation showed a tendency to have higher masticatory forces than N_IF, E_IF, and F_IF under simulation of chewing food. As a result, the curves in Figure 6 showed that there was no significant difference between skulls with food and without food in the average range of human masticatory force.

### 3.2 Stress distributions

**Figure 7:**
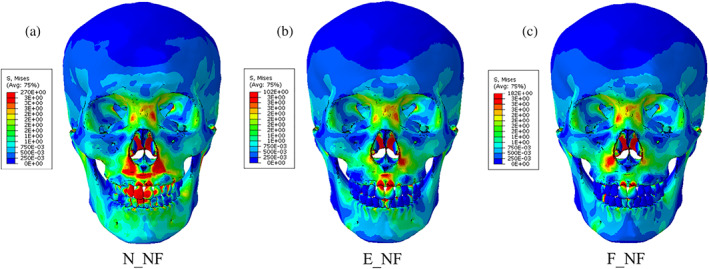
Stress distributions of the skull models without food

The second assessment was von Mises stress distribution of the skull. Figure 7 illustrates the stress profiles of skull models without food, and Table 3 presents the stress profiles of the full skull and the molar parts including food, and the average stress values at molar roots, which are obtained from the skull models with food. The pictures were captured at an occlusal force level of about 250 N, the average human masticatory force (Kwon, Yoo, Kwon, & Kim, 2006). The stress values in Table 3 were calculated as the average of von Mises stress components at integration point of each element, which was selected in molar roots.

**Table 3:** Comparison of stresses of the skull models including food

Model	Stress distribution		Average stress value of roots (MPa)				
	Full skull	Molars and food					
N_IF 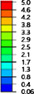	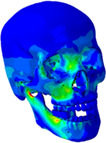	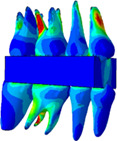	Teeth	#14	#15	#16	#17
			Stress	2.0	1.6	2.4	1.4
			Teeth	#44	#45	#46	#47
			Stress	0.8	0.7	2.1	0.7
E_IF 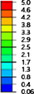	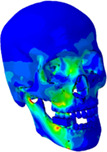	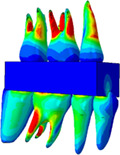	Teeth	#14	#15	#16	#17
			Stress	NA	2.7	4.9	2.4
			Teeth	#44	#45	#46	#47
			Stress	0.8	1.0	2.8	1.0
F_IF 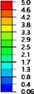	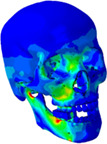	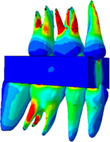	Teeth	#14	#15	#16	#17
			Stress	NA	2.6	4.4	2.2
			Teeth	#44	#45	#46	#47
			Stress	1.1	1.0	2.8	0.9

Stress distributions of the skulls without food generally show a comparable tendency regardless of occlusal relationships. As shown in Figure 7, relatively high stresses are observed in nasal bone and periphery of nasal cavity, and zygomatic bone and frontal bone have almost same stress profiles.

In the cases of the skulls with food (Table 3), stress propagation of the full skull was generally similar in all three cases. Stress was distributed from the loading points of the mandibular surface to the mandibular body, ramus, and condyle. Notably, it was shown that stress was concentrated in the mandibular notch, where the cranium tied with the TMJ disc, and in the alveolar bone around mandibular molars. In the maxilla, stresses were distributed in the areas of the frontal process, zygomatic bone, nasal bone, lacrimal bone, and orbital plate, but it was not significantly observed from frontal bone or the left side of the skulls. It was predicted that stresses primarily occur on the side of chewing food and propagated from the mandible to the maxilla.

The relatively high stresses were observed at tooth roots #16 and #46 in all the cases although stress concentration was different between normal occlusion; N_IF, and occlusions for Class II; E_IF and F_IF. As shown in stress distributions of the full skull, stresses observed from Class II occlusions were slightly higher at the alveolar bone around the lower first and second molars, and around maxillary molars. As shown in Table 3, the stress values at root #15~17 of E_IF (2.38807~4.91829 MPa), and F_IF (2.20813~4.39060 MPa) are much higher than that of N_IF (1.39675~2.35108 MPa). Moreover, the parts of molars including food showed that stresses were concentrated in roots #14, #16, and #46 only of N_IF, while the stresses distributed on relatively large area of all the roots (#15~17 and #44~47) of E_IF and F_IF. Since the models of E_IF and F_IF had fewer teeth to bear masticatory force than N_IF due to tooth extraction, the remained teeth in the models with Class II occlusions happened to be subjected to stresses larger than the model with normal occlusion.

## 4 DISCUSSION

### 4.1 Experiment for validation of the analytical methodology

**Figure 8:**
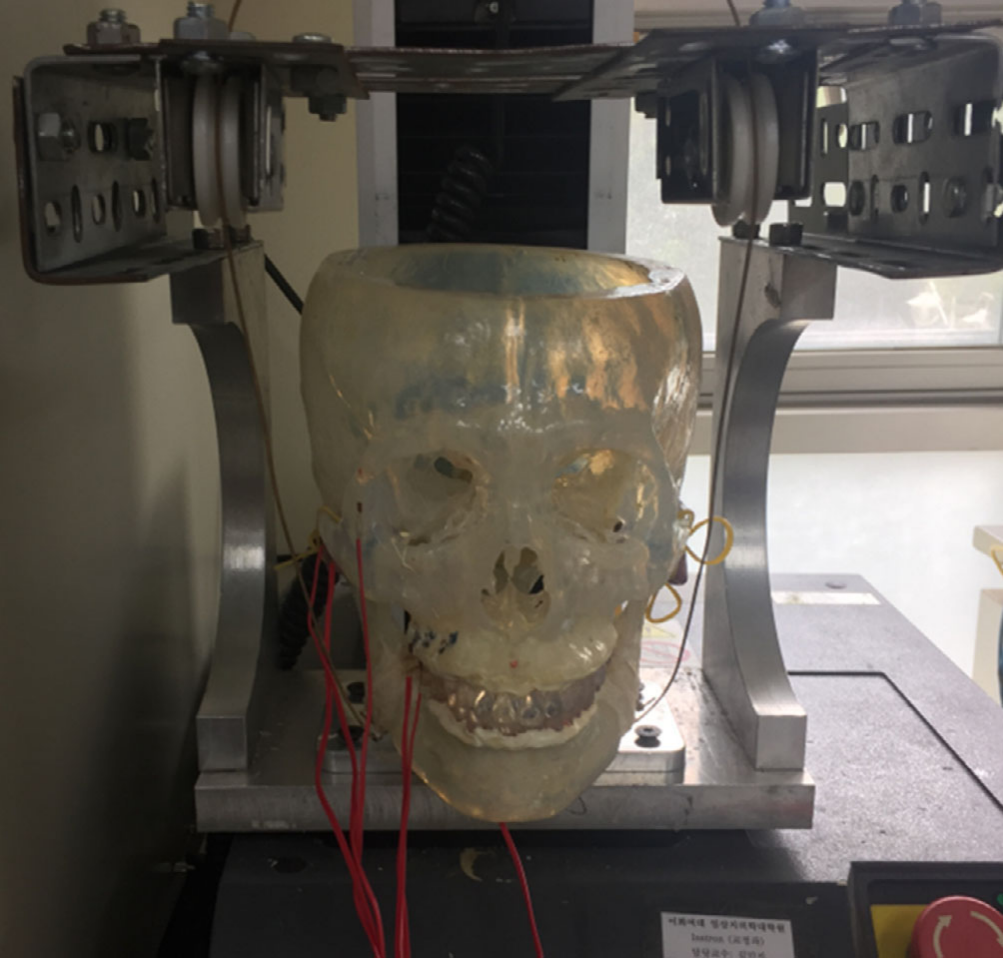
Experimental setup (Lee et al., 2017)

In general, most analytical studies of the human body are compared to in vivo or in vitro tests for validation. However, it is difficult to ensure the safety of participants in experimental testing related to dental biomechanical research due to the limited area accessible for data measurement. In previous studies (Lee et al., 2016, 2017), replica skull specimens were fabricated based on the CT images, which were used for FE models, and occluding teeth tests were conducted. As shown in Figure 8, strain gauges were attached to selective locations on the skull and teeth, such as the buccal and palatal surface of molars, the frontozygomatic suture on the lateral orbital rim, and the zygomatic process of the temporal bone. Both experimental and FE analytical results showed a similar tendency; strains at the palatal surface of molars were the highest, while those at the buccal surface of molars were the lowest. Furthermore, strains that occurred during testing were generally 6~7 times bigger than those predicted from the FE analyses. The difference of strains is similar to the difference in material properties, because photopolymer resin (TSR‐821) used for the experimental specimen has an elastic modulus of 1.8 GPa, which is around 8 times lower than material models applied to the FE analysis. Even though the FE model uses material properties simplified as linear elastic and is prescribed with translational loading direction, the experimental results are in good agreement with the analytical results taking into consideration the difference in material properties. This study uses simplified and homogenized material properties, and prescribes loading in translational direction. For the more accurate simulation of human masticatory action and the observation in microlevel, current modeling methods need to be improved by imposing composite material models and prescribing masticatory movement using masseter muscles, which are not within the scope of this study.

### 4.2 Evaluation of analytical result from the clinical viewpoint

**Figure 9:**
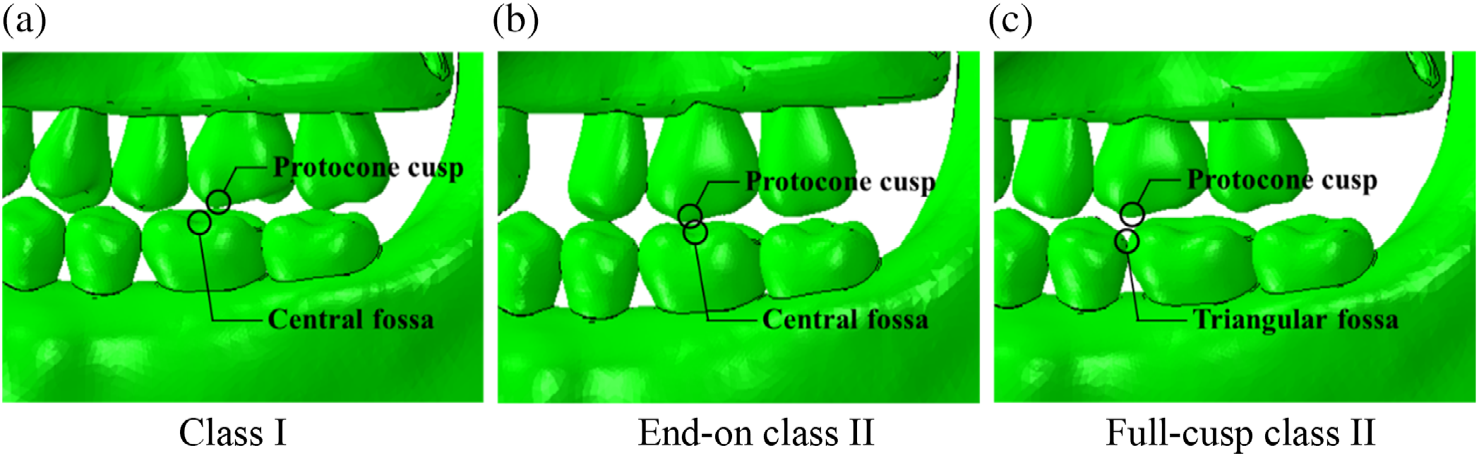
Comparison of molar relationships at the palatal side

The predicted analytical results need to be reasonable from a clinical perspective related to post‐orthodontic treatment cases, not only structural and mechanical viewpoints. Comparing the results among the FE models without food in Figure 5, the bite force–mandible movement curve predicted from the model of E_NF was close to the curve from N_NF, more than the curve from F_NF. In practice, the occlusal relationship of full‐cusp Class II is aesthetically better since there is no embrasure between teeth. However, it is theoretically explained that occlusion for full‐cusp Class II shows slightly worse masticatory function and efficiency than occlusion for end‐on Class II. Jang, Kim, and Chun (2012) reported that cusp‐to‐central fossa relationships were observed in the majority of occlusions for Class I (89.6%) and end‐on Class II (86.7%), while lingual cusp‐to‐mesial triangular fossa or a marginal ridge relationship was observed in full‐cusp Class II occlusions. Therefore, occlusions for Class I and end‐on Class II have similar mean occlusal contact areas, whereas full‐cusp Class II occlusion has significantly lower values. Lee, Kim, and Chun (2015) also stated Class I molar relationship finishing exhibits a greater area of contact than full‐cusp Class II finishing. As shown in Figure 9, the protocone cusp, the largest cusp of the maxillary first molar met the mesial‐triangular fossa of lower first molar and the distal‐triangular fossa of the lower second premolar during occluding teeth in a full‐cusp Class II relationship, while it was put into the central fossa of the mandibular first molar in Class I and end‐on Class II occlusions. The protocone cusp acts as a pestle and the central fossa functions as a socket. Since the triangular fossa cannot function properly as a socket due to its relatively even surface, occlusion for full‐cusp Class II has smaller occlusal contact areas.

However, many dentists suggest that the difference between the cases in end‐on Class II and full‐cusp Class II is not significant from a clinical point of view (Koo et al., 2017). Mastication activity is divided into two phases; Phase 1 (incursive) and 2 (excursive) (Hiemae & Kay, 1972; Kay & Hiemae, 1974). Food is punctured and crushed by molar cusps in Phase 1, and the food processing is described as grinding with an anterior‐medial lower jaw movement in Phase 2. That is, chewing food occurs mainly during Phase 1. Benazzi, Kullmer, Grosse, and Weber (2011) identified the occlusal contact area in lower first molar through collision detection algorithms during occluding teeth simulations. The results show that the contact points are mostly the buccal and lingual marginal ridges in Phase 1. Therefore, it can be predicted that the marginal ridge of the molar performs in a significant role during chewing food, instead of the central fossa in occluding teeth. In this study, the curves of masticatory force–mandible movement from E_IF and F_IF were approximately equal (Figures 5 and 6) and stress distribution of E_IF and F_IF, especially the parts of molars, were very similar to each other (Figure 7). Considering that chewing food is a more common behavior than occluding teeth, it is also predicted from the model that the occlusal relationships of end‐on Class II and full‐cusp Class II can be considered to have no difference from a clinical as well as a structural and mechanical viewpoint. Even when compared with stresses on maxillary and mandibular first molars (#16 and #46), which are primary teeth for mastication (Martinez Choy et al., 2017), relatively high stresses are observed at tooth #16 and #46 in Class I and Class II occlusions. This explains mechanically how orthodontic treatment with teeth extraction can result in similar masticatory function to the normal occlusion.

### 4.3 Assessment of the effects of the material property of food

A parametric study was carried out to identify the effect of food on structural behaviors of the skull. Two types of food were selected taking into consideration the variation of elastic moduli; the original food model was candy, which had an elastic modulus of 120.17 MPa and the new food model was a carrot, which had an elastic modulus of 7.34 MPa (Figure 4). FE analyses applying each material model were performed under the same conditions.

**Figure 10:**
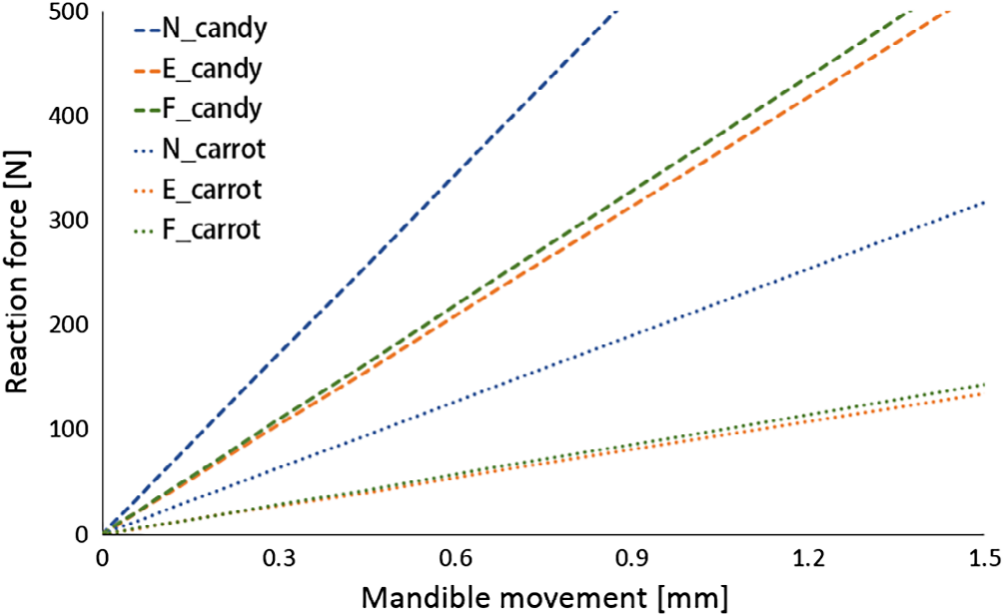
Force–displacement relationships obtained from the parametric study

Figure 10 illustrates the relationships between the masticatory force and mandible movement from the parametric study. Regardless of the type of food, the skull with a Class I occlusion showed the highest masticatory force, followed by full‐cusp Class II, and end‐on Class II. According to the order of material properties, a skull chewing candy shows a greater masticatory force than a skull chewing carrot.

The elastic modulus of candy (120.7 MPa) was around 16.44 times larger than that of carrot (7.34 MPa). The slope of the curve from N_candy was approximately 2.7 times higher than that from N_carrot, and the slopes of the curves from E_candy and F_candy were about 3.8 times higher than those from E_carrot and F_carrot. Therefore, we predict that the masticatory force when chewing harder and stiffer food increases, but this increased amount is not proportional to the difference in the material properties of food.

## 5 CONCLUSIONS

The relationship between masticatory force and mandible movement showed that there was no significant difference between skulls with food and without food in the range of masticatory force. In all the cases of the skull including food, stresses were similarly propagated and distributed, and the highest level of stress was observed at roots #14 and #46. Skulls with end‐on Class II and full‐cusp Class II occlusal relationships showed very similar masticatory forces and stress distributions, which demonstrates that the cases of end‐on and full‐cusp Class II can be considered to have no significant difference from clinical as well as mechanical viewpoints. The masticatory force increased when chewing harder and stiffer food comparing skulls including different food models, but the relation with differences in material properties was not linearly proportional.

## Supporting information


**Supporting Information File001**
