# Erratum

**DOI:** 10.1002/cre2.424

**Published:** 2021-04-04

In the following article,^1^ two author names (Hee‐Sun Kim and Jae‐Yong Park) were not included in the author byline. The correct author list is:

Hee‐Sun Kim^1^ | Jae‐Yong Park^1^ | Yeo‐Kyeong Lee^1^ | Youn‐Sic Chun^2^.

^1^Architectural and Urban System Engineering, Division of Sustainable Systems Engineering, ELTEC College of Engineering, Ewha Womans University, Seoul, South Korea.

^2^Orthodontics & dentofacial orthopedics, School of Medicine, Ewha Womans University, Seoul, South Korea.

We apologise for this error.
