# Supplementary material for: An investigation into structural behaviors of skulls chewing food in different occlusal relationships using FEM
Source: Clin Exp Dent Res. 2019 Dec 20;6(3):277–85. doi: 10.1002/cre2.273 (PMC7301394; doi:10.1002/cre2.273)
Supplement: Supplementary file 1 — Supporting Information File001 [file CRE2-6-277-s001.docx]

The authors wish to thank the reviewer for the valuable comments and remarks. The responses are as follows. The revised parts were also highlighted in the manuscript.

**Reviewer’s comments and responses:**

**I think that this manuscript is an interesting study of the effect of different occlusal relationships on structural and mechanical behaviors of skulls through the simulation of chewing on food. However, I think there are some items to be improve.**

**1. Abstract: P3, L 14-16**

It is predicted that there is no significant structural problem according to orthodontic procedure with teeth extraction.

**Comment : I think that you couldn’t enough explain for structural problem of orthodontic procedure with teeth extraction from just the results.**

**You should add explain or rephrase.**

→ The authors tried to mention that there is no significant difference of structural and mechanical behaviors between end-on class II and full-cusp class II models. In this paper, the structural and mechanical behaviors denote bite force-mandible movement relationships and stress distributions of skull and teeth. Since the sentence in the current version can mislead the authors’ intention, it is modified as following.

“It is predicted that there is no significant difference of bite force-mandible movement relationships and stress distributions of skull and teeth, between end-on class II and full-cusp class II models.”

**2. Abstract: P3, L 16-18**

From clinical as well as mechanical viewpoints, occlusal relationships of end-on class II and full-cusp class II can be considered as no difference in mastication, regardless of food types.

**Comment : I think that you didn’t enough explain because you used just two foods (candy and carrot). You should rephrase this sentence.**

→ The authors agree that the predicted food models are limited to candy and carrot cases. Therefore, the authors revised sentence to clarify such limitation as following.

“When simulating chewing activities on candy and carrot, it is also found that there is no difference of masticatory performance between class II occlusions, from structural as well as mechanical perspectives.”

**3. Summary: P5, L 4,5 Methods**

Finite element (FE) skull models of occlusion for class I, end-on class II, and full- cusp class II were generated considering general post-orthodontic treatment cases.

**Comment : I think that [end-on classⅡ] isn’t include general post-orthodontic treatment.**

**You should rephrase this sentence.**

→ The authors agreed to the reviewer’s comment and modified the sentence as following.

“Finite element (FE) skull models of occlusion for class I, end-on class II, and full- cusp class II were generated. End-on class II and full-cusp class II were chosen as mild and severe class II occlusions, respectively.”

**4. Summary: P5, L 17-20 Conclusion**

We predict that there is no significant structural problem according to orthodontic procedure with teeth extraction. From clinical as well as mechanical viewpoints, occlusal relationships of end-on class II and full-cusp class II can be considered to have no mastication difference, regardless of food type.

**Comment : I think that the aim of this study was the effect of different occlusal relationships on skull structural and mechanical behaviors through simulation of chewing food. However, this conclusion have described methodology of orthodontic treatment and food type.**

**You should change sentence of purpose or conclusion.**

→ The authors revised the summary in order to have conclusion consistent with the aim of the study.

“When simulating chewing activities on candy and carrot, it is also found that there is no difference of masticatory performance between class II occlusions, from structural as well as mechanical perspectives.”

**5. Introduction: P6, L 2,3**

teeth orthodontic patients →orthodontic patients

**I think that you should delete [teeth], because this journal is for Dentist.**

→ Authors corrected the terminology according to the reviewer’s comment. Appreciated for the pointing out.

**6. Results: P10, L 13-15**

Moreover, the parts of molars and food showed that stress was concentrated in all the roots (#15~17 and 44~47) of E_IF and F_IF, while only roots #14, 16, and 46 of N_IF.

**Comment : Is that showed for red marker at Fig.7? If so, I seem that 44,45 and 47 of EIF and FIF have not showed concentrated stress.**

→ The criteria defining red colored area was arbitrarily determined as 5MPa. Therefore, the authors tried to mention that the stresses over 5MPa were concentrated in roots #14, 16, and 46 only for the normal occlusion case, while the stresses were distributed on relatively large area of all the roots (#15~17 and 44~47) of the class II models. In order to clarify, the author added the stress values at molar roots, and modified the sentence as following.

“As shown in Table 3, the stress values at root #15~17 of E_IF (2.38807~4.91829 MPa) and F_IF (2.20813~4.39060 MPa) are much higher than that of N_IF (1.39675~2.35108 MPa). Moreover, the parts of molars including food showed that stresses were concentrated in roots #14, 16, and 46 only of N_IF, while the stresses distributed on relatively large area of all the roots (#15~17 and 44~47) of E_IF and F_IF. Since the models of E_IF and F_IF had less teeth to bear masticatory force than N_IF due to tooth extraction, the remained teeth in the models with class II occlusions happened to be subjected to stresses larger than the model with normal occlusion.”

**7. Results: P10, L 15-16**

It can be predicted that the load subjected to root #14 was distributed to neighboring teeth because tooth #14 was removed in the E_IF and F_IF models.

**Comment : There was simulated for post orthodontic treatment, thus you have to set up #13 at extracted #14 space in the E_IF and F_IF models. Or you should describe other consideration. I think that the consideration isn’t clinical view.**

→ When generating models with class II occlusions, the authors removed tooth #14 part from the skull model and moved #13 at the removed space of #14 as the reviewer pointed out. Therefore, the class II models consisted of less teeth than class I model, which causes large stresses distributions on the remained teeth of class II models under the same masticatory force as the normal model. To clear the meaning, the authors modified the sentences as following.

“Moreover, the parts of molars including food showed that stresses were concentrated in roots #14, 16, and 46 only of N_IF, while the stresses distributed on relatively large area of all the roots (#15~17 and 44~47) of E_IF and F_IF. Since the models of E_IF and F_IF had less teeth to bear masticatory force than N_IF due to tooth extraction, the remained teeth in the models with class II occlusions happened to be subjected to stresses larger than the model with normal occlusion.”

**8. Results: P10, L 15-19**

It can be predicted that the load subjected to root #14 was distributed to neighboring teeth because tooth #14 was removed in the E_IF and F_IF models. However, maxillary and mandibular first molars (#16 and 46) were the primary teeth for mastication^30^, which explains why there was no significant structural or mechanical problems according to orthodontic treatment with teeth extraction.

**Comment : I think that you should move to Discussion this sentence in order to make it easy to understand.**

→ First sentence has been modified as responded above in comments No. 6 and 7, and the latter sentence was moved to the discussion part (section 4.2) of the revised manuscript after modified as following.

“Even when compared with stresses on maxillary and mandibular first molars (#16 and 46), which are primary teeth for mastication^30^, relatively high stresses are observed at tooth #16 and 46 in class I and class II occlusions. This explains mechanically how orthodontic treatment with teeth extraction can result in similar masticatory function to the normal occlusion.”

**9. Discussion: P11, L 15-16**

It can be predicted that E_NF is more similar to N_NF and has better occlusal contact than F_NF.

**Comment : There can to assess only reaction force by these results. I think that it is so difficult to assess occlusal contact.**

→ The authors’ intention was that the bite force-mandible movement curve predicted from the model of E_NF was close to N_NF more than F_NF, and the reason could be found from the occlusal contact. As the reviewer mentioned, Fig. 5 illustrated bite force-mandible movement relationships only. Therefore, the authors modified the sentence by removing contact part as following.

“Comparing the results among the FE models without food in Fig. 5, the bite force-mandible movement curve predicted from the model of E_NF was close to the curve from N_NF, more than the curve from F_NF.”

**10. Discussion: P11, L 27-30**

As shown in Fig. 9, the protocone cusp, the largest cusp of the maxillary first molar met the mesial-triangular fossa of lower first molar and the distal-triangular fossa of the lower second premolar during occluding teeth in a full-cusp class II relationship, while it was put into the central fossa of the mandibular first molar in class I and end-on class II occlusions.

**Comment : You didn’t describe for end-on occlusion; thus, you should add it.**

**Also, you should add describe for evidence according to protocone cusp of end-on**

**and class1 met the central fossa of the lower first molar.**

→ The authors added the picture of molar relationship from the model with end-on class II occlusion in Fig. 9 (b) as the reviewer suggested. The modified Fig. 9 is as followings.


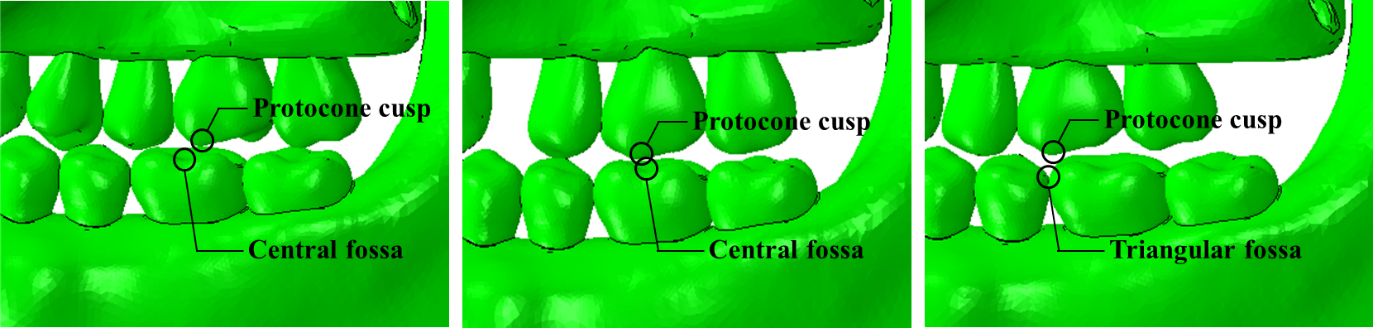


(a) Class I (b) End-on class II (c) Full-cusp class II

**Fig. 9** Comparison of molar relationships at the palatal side

**11. Discussion: P12, L 26,27**

The class II curves were very similar to each other in all cases.

**Comment : In Fig.5 and 6, You have described the masticatory force-mandible movement curve from E_NF was closer to N_NF than the curve from F_NF. However, in this sentence, you have shown the class II curves were very similar to each other in all cases. You should explain this difference in order to make it easy to understand.**

→ The authors tried to explain about Fig. 10, such that the predicted bite force-mandible movement relationships of the model with end-on class II were close to the those of full-cusp class II, when chewing activities on carrot and candy were simulated. Therefore, ‘in all cases’ actually denoted ‘chewing candy and carrot simulations’, not ‘clenching and food chewing simulations’. Nonetheless, because the very sentence that the reviewer pointed out might be confusing, the authors decided to delete it. Still, there are sentences that compares masticatory forces of the models with end-on class II and full-cusp class II under chewing carrot and candy in the manuscript as following.

“Figure 10 illustrates the relationships between the masticatory force and mandible movement from the parametric study. Regardless of the type of food, the skull with a class I occlusion showed the highest masticatory force, followed by full-cusp class II, and end-on class II. According to the order of material properties, a skull chewing candy shows a greater masticatory force than a skull chewing carrot.”

**12. Conclusion: P13, L 8-9**

In all the cases of the skull including food, stresses were similarly propagated and distributed, and the highest level of stress was observed at roots #14 and #46.

**Comment : Have you explained at Results this sentence? If no, you should add. Moreover, there might be difficult to understand for reader by only Fig.7. You should fix Fig.7 or add some Table in order to make it easy to understand.**

→ In the section of Results (section 3.2), the authors explained the stress distributions of the skull with class I, end-on class II and full-cusp class II, such that “In the cases of the skulls with food (Table 3), stress propagation of the full skull was generally similar in all three cases.” and “The relatively high stresses were observed at tooth roots #16 and 46 in all the cases.”

To show the stress values at molar roots, the authors fixed the figure by changing Fig.7 into Table 3, and added the sentence to compare the stress at root #16 in the revised manuscript as following.

“As shown in Table 3, the stress values at root #15~17 of E_IF (2.38807~4.91829 MPa) and F_IF (2.20813~4.39060 MPa) are much higher than that of N_IF (1.39675~2.35108 MPa). Moreover, the parts of molars including food showed that stresses were concentrated in roots #14, 16, and 46 only of N_IF, while the stresses distributed on relatively large area of all the roots (#15~17 and 44~47) of E_IF and F_IF. Since the models of E_IF and F_IF had less teeth to bear masticatory force than N_IF due to tooth extraction, the remained teeth in the models with class II occlusions happened to be subjected to stresses larger than the model with normal occlusion.”

**Table 3.** Von Mises stress distributions of the skull models including food

| Model | Stress distribution | | Average stress value of roots  [MPa] | | | | |
| --- | --- | --- | --- | --- | --- | --- | --- |
|  | Full skull | Molars and food |  |  |  |  |  |
| N_IF   | 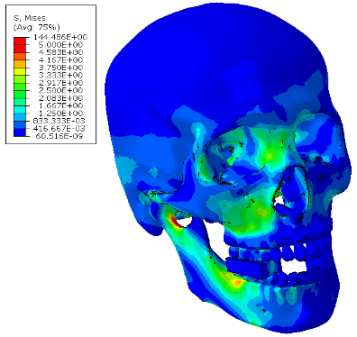 | 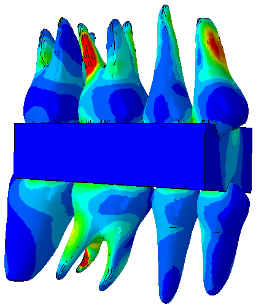 | Teeth | #14 | #15 | #16 | #17 |
|  |  |  | Stress | 2.0 | 1.6 | 2.4 | 1.4 |
|  |  |  | Teeth | #44 | #45 | #46 | #47 |
|  |  |  | Stress | 0.8 | 0.7 | 2.1 | 0.7 |
| E_IF   | 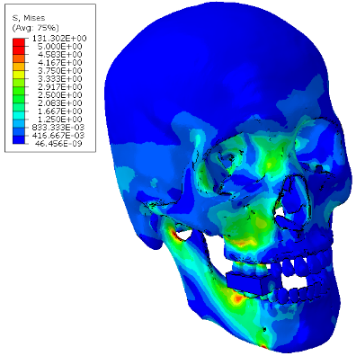 | 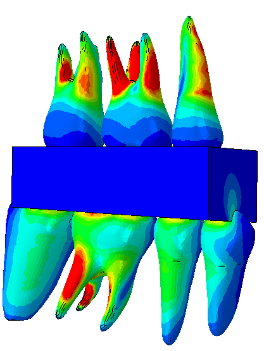 | Teeth | #14 | #15 | #16 | #17 |
|  |  |  | Stress | NA | 2.7 | 4.9 | 2.4 |
|  |  |  | Teeth | #44 | #45 | #46 | #47 |
|  |  |  | Stress | 0.8 | 1.0 | 2.8 | 1.0 |
| F_IF   | 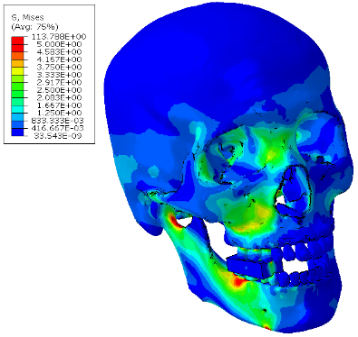 | 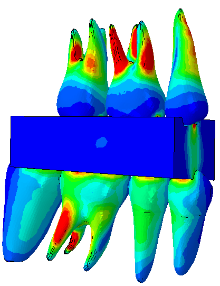 | Teeth | #14 | #15 | #16 | #17 |
|  |  |  | Stress | NA | 2.6 | 4.4 | 2.2 |
|  |  |  | Teeth | #44 | #45 | #46 | #47 |
|  |  |  | Stress | 1.1 | 1.0 | 2.8 | 0.9 |

**13. Conclusion: P13, L 16-20**

This FE simulation has several limitations. The skull models were simplified in terms of material properties, contact, and boundary conditions. In further studies, these limits are going to be overcome for more accurate simulation by using micro-level composite material models and applying other masticatory directions; mesial-distal and buccallingual, which were not within the scope of this study.

**Comment : I think that you should move to Discussion this sentence.**

→ The authors agreed to the reviewer’s comment and moved to the discussion part (in the end of section 4.1) of the revised manuscript as following.

“This study uses simplified and homogenized material properties, and prescribes loading in translational direction. For the more accurate simulation of human masticatory action and the observation in micro-level, current modeling methods need to be improved by imposing composite material models and prescribing masticatory movement using masseter muscles, which are not within the scope of this study.”

**14. Fig.7: P23**

**Comment : Do you have the data that stress distributions of the skull models without food? If so, I think that you should show the data in order to make it easy to understand.**

→ The authors added the stress distributions of the skull models without food (Figure 7) as the reviewer suggested. The added the figure and the explanation are as followings.

“The second assessment was von Mises stress distribution of the skull. Figure 7 illustrates the stress profiles of skull models without food, and Table 3 presents the stress profiles of the full skull and the molar parts including food, and the average stress values at molar roots, which are obtained from the skull models with food. The pictures were captured at an occlusal force level of about 250N, the average human masticatory force^31^. The stress values in Table 7 were calculated as the average of von Mises stress components at integration point of each element, which was selected in molar roots.

Stress distributions of the skulls without food generally show a comparable tendency regardless of occlusal relationships. As shown in Fig. 7, relatively high stresses are observed in nasal bone and periphery of nasal cavity, and zygomatic bone and frontal bone have almost same stress profiles.”


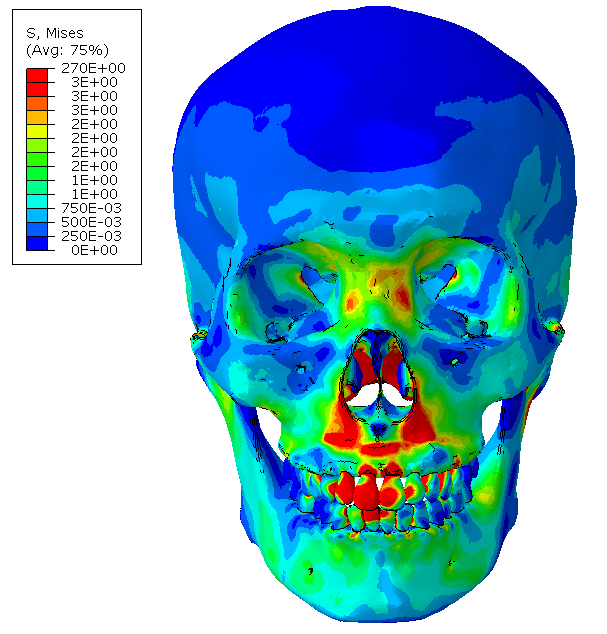


1. N_NF


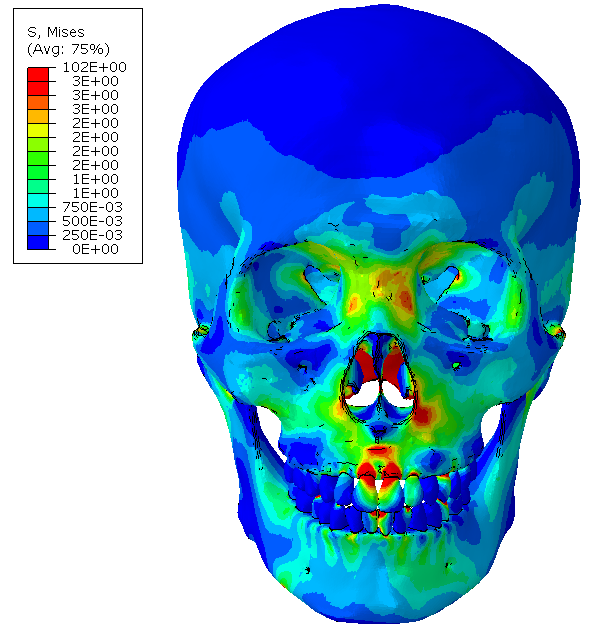


1. E_NF


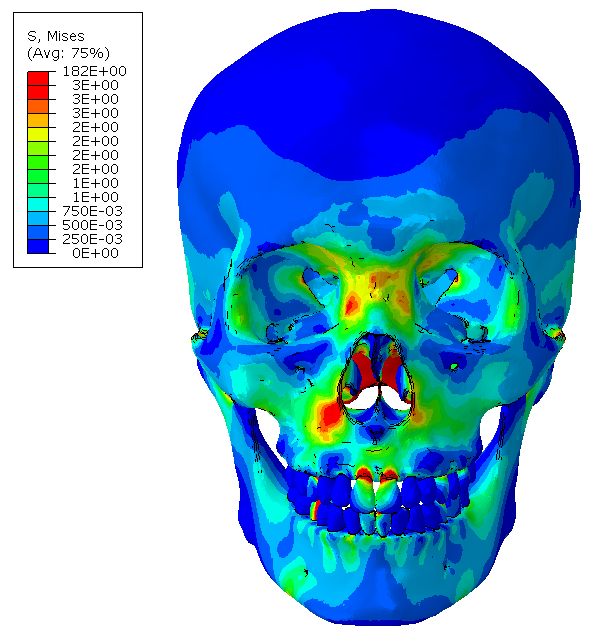


1. F_NF

**Fig. 7** Stress distributions of the skull models without food
